# A randomized phase II clinical trial of dendritic cell vaccination following complete resection of colon cancer liver metastasis

**DOI:** 10.1186/s40425-018-0405-z

**Published:** 2018-09-29

**Authors:** Javier Rodriguez, Eduardo Castañón, Jose Luis Perez-Gracia, Inmaculada Rodriguez, Antonio Viudez, Carlos Alfaro, Carmen Oñate, Guiomar Perez, Fernando Rotellar, Susana Inogés, Ascensión López-Diaz de Cerio, Leyre Resano, Mariano Ponz-Sarvise, Maria E. Rodriguez-Ruiz, Ana Chopitea, Ruth Vera, Ignacio Melero

**Affiliations:** 1 Clinica Universidad de Navarra, Avenida Pio XII, 36, 31008 Pamplona, Spain; 2 Complejo Hospitalario de Navarra, Avenida Irunlarrea 5, 31008 Pamplona, Spain; 3 Centro de Investigacion Medica Aplicada, CIMA, Avenida Pio XII, 36, 31008 Pamplona, Spain; 4 CIBERONC, Madrid, Spain; 5 Instituto de investigación de Navarra, IDISNA, Pamplona, Spain; 6 Pamplona, Spain

**Keywords:** Dendritic cell, Colon cancer, Vaccine, Relapse prevention, Randomized clinical trial

## Abstract

Surgically resectable synchronic and metachronic liver metastases of colon cancer have high risk of relapse in spite of standard-of-care neoadjuvant and adjuvant chemotherapy regimens. Dendritic cell vaccines loaded with autologous tumor lysates were tested for their potential to avoid or delay disease relapses (NCT01348256). Patients with surgically amenable liver metastasis of colon adenocarcinoma (*n* = 19) were included and underwent neoadjuvant chemotherapy, surgery and adjuvant chemotherapy. Fifteen patients with disease-free resection margins were randomized 1:1 to receive two courses of four daily doses of dendritic cell intradermal vaccinations versus observation. The trial had been originally designed to include 56 patients but was curtailed due to budgetary restrictions. Follow-up of the patients indicates a clear tendency to fewer and later relapses in the vaccine arm (median disease free survival –DFS-) 25.26 months, 95% CI 8.74-n.r) versus observation arm (median DFS 9.53 months, 95% CI 5.32–18.88).

**Electronic supplementary material:**

The online version of this article (10.1186/s40425-018-0405-z) contains supplementary material, which is available to authorized users.

## Introduction

In spite of high expectations, cancer vaccines have found little applicability in oncology clinical practice [1]. It is likely that vaccines will be more efficacious at preventing relapse in patients with minimal residual disease status as opposed to advanced cases with bulky and immunosuppressive disease [1, 2].

Dendritic cell vaccines harness the antigen-presenting functions of these leukocyte subsets to induce antitumor CD8 and CD4 T cell responses [3]. The only exception of a FDA-approved cancer vaccine is a monocyte-derived dendritic cell product pulsed with a prostate serum antigen chimeric protein (Sipuleucel, Provenge) which was approved based on overall survival benefit [4, 5] for castration resistant metastatic prostate cancer. The nature of the antigen is likely to be important with current preference for antigens encoded by non-synonymous mutations or other gene alterations in the tumor genome [6]. Presentation of such antigens can be attained by pulsing defined peptide sequences following their genomic identification [7] or transfecting total tumor mRNA into the DCs [8]. A simpler alternative is the use of autologous tumor lysates to load the DC, which are known to immunize to at least to some extent to those relevant neoantigens [9–13].

We have developed a clinical-grade product based on autologous CD14+ monocytes differentiated to DC by means of 7-day cultures in the presence of GM-CSF and IL-4. Such cultures are incubated with autologous tumor lysate and activated by a cocktail of agents combining TNFα, poly-ICLC (Hiltonol^tm^) and IFNα [12]. We have previously reported immunogenicity and safety of the approach [2], that showed intriguing long relapse-free survival in a series of resected glioblastoma cases [14].

Colon cancer frequently metastasize to the liver and in some cases, rescue surgery is feasible [15]. Various studies recommend neoadjuvant and adjuvant chemotherapy to reduce relapse risk [16–19] that in the best series still remains over 50–60%. Previous attempts have been made to use cancer vaccines to prevent relapse, including positive trials with Bacillus Calmette Guerin (BCG) mixed with autologous irradiated tumor cells from the resection specimen. This preventive treatment to avoid relapse was effective in stage II colon carcinoma but not in stage III or IV [20].

In this disease scenario of surgically amenable colon cancer with liver metastases, minimal residual disease is expected following surgery plus chemotherapy. We have tested our previously reported DC formulations loaded with autologous tumor lysates [12] in a randomized fashion. Even if the trial had to be halted with only 15 randomized patients, our follow-up observation strongly suggests a beneficial effect of the vaccination scheme on disease-free survival (DFS).

## Patients and methods

### Patient selection and treatment

This is an open label randomized phase II trial (Study With Dendritic Cell Immunotherapy in Resected Hepatic Metastasis of Colorectal Carcinoma registered on May 11th 2011 in https://www.clinicaltrials.gov/ct2/show/NCT01348256) evaluating the efficacy of dendritic cell vaccination versus observation in patients with potentially resectable liver metastases from colon cancer who underwent a complete scheme of neoadjuvant chemotherapy, surgery and adjuvant chemotherapy. The investigative product dossier (IMPD), the clinical trial protocol were approved by the Agencia Española del Medicamento y productos saninatios (AEMPS). The clinical trial protocol and informed consent forms were approved by the regional ethics committee.

General inclusion criteria comprised patients diagnosed with stage IV colon cancer with liver metastases, ECOG <=2, hemoglobin > 9 g/dl, platelets > 50.000/mm3, leucocytes > 3000/mm3, Bilirubin < 5 x ULN, ASAT and ALAT < 5 x ULN, Creatinine < 2 x ULN and negativity to HBV, HCV and HIV. Patients should have had tumor sample available in order to produce autologous tumor lysate for DC loading. Exclusion criteria included active infection or conditions which could jeopardize patient’s safety, concurrent treatment for the oncological disease (either approved or investigational), active CNS metastases, second malignancies excluding squamous or basal cell carcinoma, cervical carcinoma or other tumors treated radically within the previous 3 years, pregnant or breastfeeding women and patients receiving immunosuppressive agents.

Patients should have undergone a scheme of neoadjuvant chemotherapy (including standard 5FU + platinum based schemes), followed by surgery of liver metastases and the primary tumor (if existing) as well as an adjuvant chemotherapy scheme (including 5FU+ platinum based regimens).

### Dendritic cell preparation and vaccination

In the surgical procedure, enough malignant tissue material had to be retrieved to prepare tumor lysate by freeze/thaw with a first cycle of heating at 90 °C for five minutes to favor protein aggregation and inactivate proteases. In the DC treatment group, patients underwent 2–3 volume leukoapheresis and CD14+ monocytes were selected by clinical-grade immunomagnetic selection using clinicimacs technology (Miltenyi biotec). As previously described [2], monocytes were cultured in cell-culture flasks (175 cm2; Corning, Sigma- Aldrich, St. Louis, MO) for 7 days in AIM-V serum-free media (Life Technologies-BRL, Gaithersburg, MD) supplemented with GM-CSF (1000 U/ml; Leukine, Berlex, Richmond, CA) and IL-4 (500 U/ml; R&D Systems, Minneapolis, MN). With this protocol, at least over 90% CD11c + viable dendritic cells were obtained in all cases.

Tumor lysates were generated from needle-core tumor biopsies or surgical samples. Tumor tissue disruption was performed with the GentleMacs dissociator device (Miltenyi Biotec), followed by the freezing/thawing and irradiation procedures, to be be subsequently cryopreserved at 220 °C until used.

DC cultures were pulsed with lysate at 100 micrograms of protein/mL and two hours later induced to mature for 24 h with polyICLC (Hiltonol) 1 μg/mL, TNFα 50 ng/ml (Boehringer Ingelheim, Ingelheim, Germany) and IFNα (1000 IU/ml; Schering-Plough, Kenilworth, NJ). Cells were immediately used or frozen for subsequent administrations to patients.

Freezing and thawing of matured and Ag-loaded DC were performed, as described previously [11]. DC were slowly frozen in autologous serum with 5% *v*/v DMSO by using a computer-assisted step down freezer (CM-25; Carburos Metalicos). The first two treatments were performed with cultured cells without any previous freezing step, whereas the rest of the treatments were prepared with thawed DC.

DC activation/maturation was confirmed by FACS assessing increases in the immunofluorescence of CD80, CD86, and HLA-DR. Immediately after thawing, cell viability was assessed by trypan blue exclusion ranging from 76 to 98%. Flow cytometry analysis was performed at day 7 using FACScan (BD Biosciences, San Diego, CA). Release criteria for DC included 75% HLA-DR+ and CD11c bright and negative tests for microbial contamination. All the vaccines have passed the release criteria. Repeated Vaccination was performed by intradermal injection given bilaterally alternating the anterior upper thigh regions of the patients.

### Flow cytometry

For DC phenotype characterization, the following Abs were used: CD11c (clone 3.9 from Biolegend), HLA-DR (clone G46–6 from Biolegend), CD80 (clone L307.4 from Pharmingen), CD86 (clone 2331(FUN-1) from Pharmingen) and CD14 (clone M5E2 from Biolegend). Samples were analyzed using a FACSCanto flow cytometer (BD Biosciences).

### Cytokine determinations

Simultaneous measurement of TNF-α, IL-1β, IL-6, IL-8, IL-10 and IL-12p70 in the supernatant from the mature DCs was analyzed by microparticle-based flow cytometry (Cytometric Bead Array) according to the manufacturer’s instructions (BD Biosciences, San Jose, CA).

### IFNγ ELISPOT

Human IFNγ ELISPOT PRO Kit (MABTECH) was used according to manufacturer instructions. Plates were blocked with RPMI 1640 supplemented with 10% FBS for 1 h at 37 °C. The medium was aspirated, and effector cells (2.5 × 10^5^) were seeded in triplicates in RPMI 1640 with 10% heat-inactivated FCS. PBMC isolated before treatment and at day + 30 post treatment, were used as effector cells. Stimulator cells were autologous mature DC loaded with tumor lysates (50–250 μg/ml). DC had been matured with TNF-α (50 ng/ml), IFN-α (1000 U/ml), and poly(ICLC) (20 μg/ml) for 48 h in AIM-V medium (BioWhittaker Lonza). DCs (5 × 10^4^) were cultured with the effector cells. Negative control wells contained equally seeded with unloaded DC and without DC. Positive controls were standard SEB 1:100 dilution (SIGMA). Cells were incubated at 37 °C in 5% CO_2_ in a water-saturated atmosphere. After a culture period of 36 h, cells were removed by six washings with PBS/0.05% and ELISPOT was developed acording to manufacturer instructions and spots per well were automatically counted with automated immunospot counter (CTL).

### Statistical considerations

Different variables were collected including tumor size (pT), nodes invasion (pN), type of tumor margins (R0 vs R1/R2), Nagashima score, which categorizes patients regarding their suitability for liver resection taking into account number of hepatic metastatic tumors, liver metastatic tumors larger than 5 cm in diameter, resectable extra- hepatic distant metastases, regional lymph node metastases and serosal invasion of primary colorectal cancers [21], Fong score (which measures the risk of relapse after hepatic resection based on node-positive primary tumor, disease-free interval from primary to metastases < 12 months, number of hepatic lesions > 1, largest hepatic tumor > 5 cm and carcinoembryonic antigen level > 200 ng/ml) [22]. Disease-free survival was measured as the time from surgery to relapse, death or loss of follow-up. Survival curves were compared based on the Kaplan Meier estimates [23]. A log rank test was used in order to calculate the difference between curves. Median follow up was calculated based on the inverse Kaplan Meier method [24]. All calculations were performed with STATA v.14 statistical package (StataCorp 2015).

## Results

We have previously reported that a cell therapy product consisting of monocyte-derived DC loaded with heat-treated autologous tumor lysate and matured with a cocktail of poly-ICLC, TNFα and IFNα was immunogenic [11].

An unmet clinical setting in which vaccines would be very useful is the prevention of relapse following potentially curative resections of colon adenocarcinoma liver metastases. A randomized clinical was designed to address a putative beneficial effect of this treatment versus standard-of-care at our institutions.

The original design of this academically sponsored trial (NCT01348256) included 56 patients but budget restrictions forced an early termination of recruitment, when only 19 patients had signed informed consent.

**Fig. 1:**
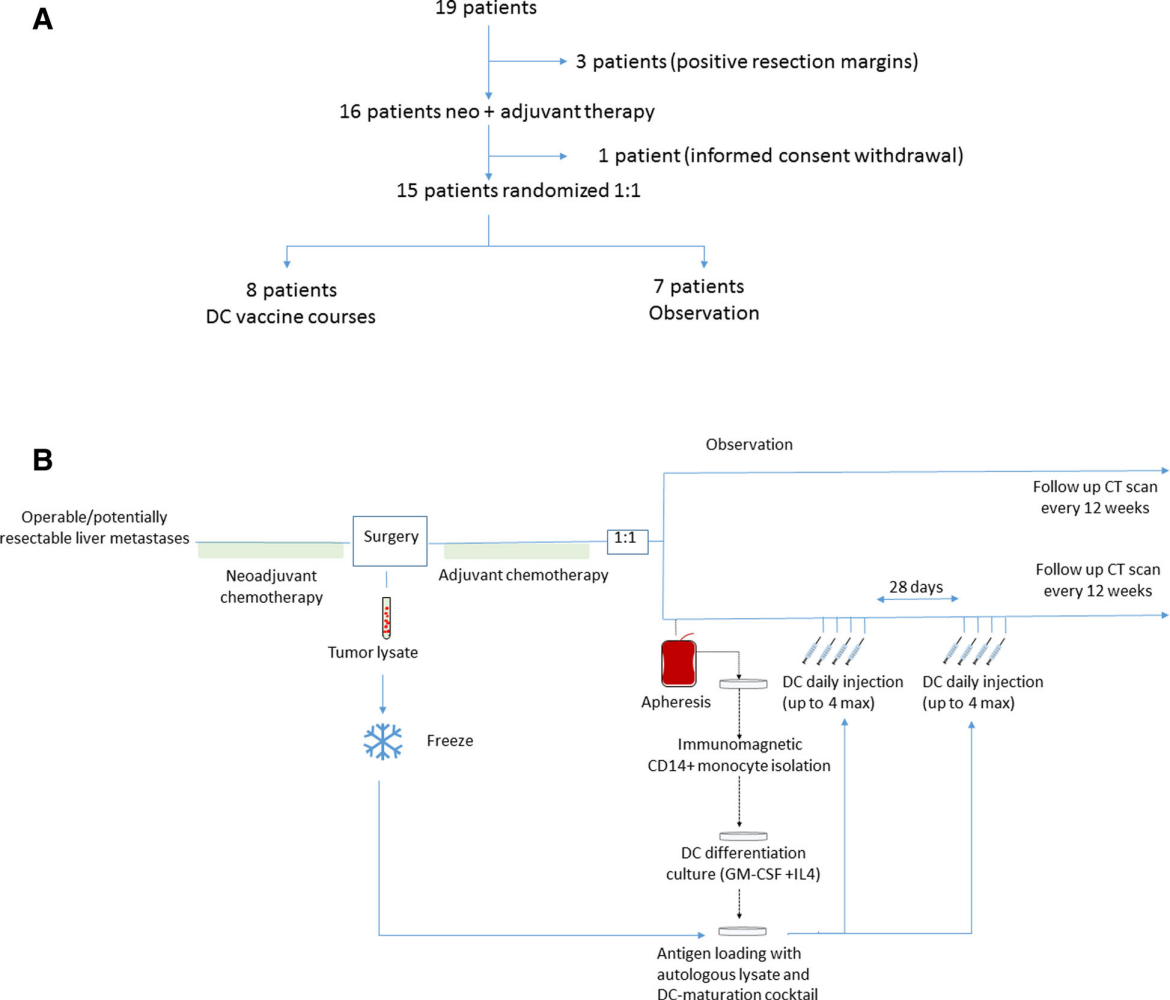
Clinical trial design and treatment generation scheme. **a** Graphical summary of patient accrual, randomization and treatment. **b** Schematic representation of production of dendritic-cell vaccines loaded with autologous tumor lysate in patients undergoing complete surgical resection of colon cancer liver metastasis

As indicated in Fig. 1a, three of the patients were excluded for evaluation due to positive resection margins (>R0) following neoadjuvant chemotherapy and surgery. One patient withdrew informed consent after neoadjuvant treatment, and thus was not randomized. Neoadjuvant chemotherapy consisted of standard chemotherapy 5-FU-based cycles (Table 1) .

**Table 1:** Patient population description

	Dendritics cell vaccine (*n* = 8)	Observation (*n* = 7)
Age (mean, CI 95%)	58.75 (52.25–65.25)	56.29 (52.92–69.66)
Sex (%)		
Male	50%	62.5%
Female	50%	37.5%
ECOG (median, range)	0 (0–1)	0 (0–1)
ESMO (median, range)	1 (0–1)	0 (0–1)
Köhne (median, range)	0 (0–1)	0 (0)
Nagashima score (%)		
Low risk	0%	50%
Moderate risk	33.33%	50%
High risk	66.67%	0%
Fong score (%)		
Low risk	50%	57.14%
High risk	50%	42.86%
Histology (%)		
Adenocarcinoma	87.5%	100%
Mucinous	12.5%	0%
CEA (%)		
< 200 ng/ml	50%	85.7%
> 200 ng/ml	50%	14.3%
Metastatic disease at diagnosis	87.5%	57.4%
Neoadjuvant cycles (mean, CI 95%)	5.75 (4.68–6.82)	6.57 (5.67–7.47)
KRAS status		
Mutant	62.5%	57.14%
Wild type	37.5%	42.6%
MSI status	37.5%	0%

Surgery included lobe or segmental liver resections as required. Once adjuvant chemotherapy was over, patients were randomized to receive dendritic cell vaccinations or observation (Fig. 1a and b).

A regimen of two cycles of four daily intradermal vaccines given bilaterally in the two upper thigh regions was followed as described [3] to maximize acute immunization.

Table 1 reflects the characteristics of the 15 patients who were in excellent performance status. Remarkably, the observation group was unbalanced with more favorable cases as depicted by risk scales (Nagashima and Fong scores). Indeed, the vaccination group had five patients with high Nagashima risk, while there was none in the observation group. All cases were diagnosed as metastatic carcinoma of the colon with more serum expression of CEA in the cases included in the vaccine arm (Table 1). Importantly, neoadjuvant chemotherapy exposure was similar in the two arms. K-RAS mutation status was well balanced between treatment and observation arms (Table 1).

Follow-up was performed every 12 weeks (range 11–14 weeks) ever since surgical treatment by contrast abdominal CT scans.

**Fig. 2:**
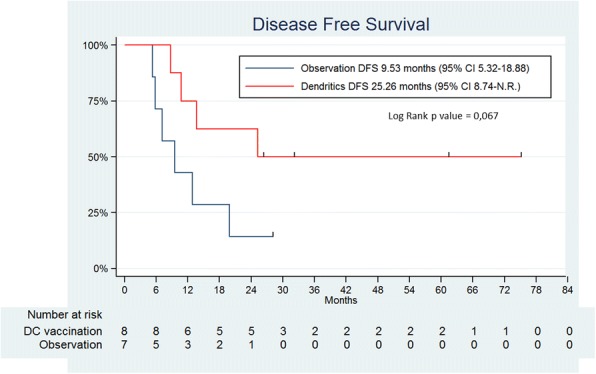
Disease-Free survival curves. Kaplan-Meier curves represent disease-free survival of vaccination versus observation arms. Ticks in the curve represent censored data. Recurrences were documented by CT-Scan and number of subjects at risk are in the table below the graphs. Probability of the difference calculated by log-rank test is given together with the estimation of median DFS of both arms

Treatment with DC was safe and only grade 1 treatment-attributable side effects were recorded. At median duration of follow-up of 42.58 months, disease free survival (DFS) appeared different in the two groups, as seen in the Kaplan-Meier curves in Fig. 2. Even though numbers of patients are small, a log-rank test estimated a probability of 0.067 and the median disease-free survival was 25.26 months in the vaccination arm as compared to 9.53 months in the observation arm.

In the vaccine arm four patients have not relapsed while four of them eventually relapsed. It is of note that there were three MSI-High cases in the vaccination arm while there was none in the observation arm (Table 1). However, two of the MSI-High cases have relapsed while one remains disease-free indicating that MSI-High status does not provide protection at the metastatic stage.

We monitored dendritic cell vaccine maturation in terms of cytokine secretion to the culture supernatant and surface expression of maturation markers (Additional file 1). DC products were highly mature and produced large amounts of Interleukin-12. As can be seen in Additional file 1, there was a tendency to larger Interleukin-8 production by DC from relapsed cases and intriguingly a higher intensity of surface expression of CD11c. However, the small number of samples precludes solid conclusions beyond hypothesis generation. IFNγ-ELISPOT assays were conducted with pretreatment (time of leukoapheresis) and postreatment (4 weeks after the second treatment cycle) samples. As shown in Additional file 2, a clear increase in reactivity to tumor lysate-loaded DC was only seen in one of the non-relapsed cases. Marginal increases were observed in other cases (Additional file 2).

## Discussion

Randomized evidence for efficacy of dendritic cell vaccination for cancer is yet missing. In cutaneous melanoma, DC pulsed with shared antigen peptides did not show evidence for benefit [25]. However, important adjuvant dendritic cell vaccination randomized trials for skin melanoma (NCT02993315) and uveal melanoma [26] are ongoing.

Our study chose resectable metastatic colon cancer because is an unmet clinical need in patients with potential minimal residual disease but at very high risk of relapse. Unfortunately, logistical/budgetary problems forced the trial to be halted with a short number of patients randomized to each of the two arms. However, these patients were kept in follow-up according to protocol and relapses evaluated as planned.

With all caveats due to patient sample size, current results are strongly supportive of a beneficial effect of DC vaccination that would warrant a confirmatory randomized clinical trial in a similar setting. It is worth mentioning that unbalanced presence of higher-risk of relapse patients (according to clinical scores) in the vaccination arm further supports the existence some degree of relapse-free survival benefit in the vaccinated arm. Reinforcing this notion, risk scales of relapse would have been predictive of higher probability of relapse in the vaccination arm. In this regard, there was also an unbalance in MSI-H status with three cases in the vaccination versus none in the observation arm. However, two of the MSI-H cases actually relapsed. This is important since MSI-H is related to less metastatic progression following surgery of the primary tumor [27] and higher antigenicity and more susceptibility to PD-1 blocking agents at metastatic stage [28]. However, once metastatic MSI-H patients show a similar progression pattern as non-MSI cases [29] as it is the case in our small series of patients.

An important question would be whether to go on with the same vaccination schedule or to provide some more boosting cycles of vaccination during the first and second year following surgery. The impression is that the vaccines may have prevented relapse in some patients, while in others vaccination might only have delayed relapse, thus advocating for extended boosting doses.

The quality and maturation of the DC product was good in relapsed and non-relapsed cases following treatment, particularly showing prominent IL-12 production. A tendency to more IL-8 production and brighter surface expression of CD11c were noted in relapsed cases, a finding that warrants confirmatory research in ongoing and subsequent clinical studies.

Other points to be considered would be to add check-point inhibitors circa vaccination dates [30] and to monitor patients for immunization against neo-antigens by IFNγ ELISPOT [12] or other techniques. Our series of IFNγ-ELISPOT assays is not sufficiently large to draw any conclusions but suggests interesting individual heterogeneity in the measurable response 4 weeks after the last vaccine.

There is recently published evidence supportive for checkpoint inhibitor immunotherapy approaches given in neoadjuvant schemes prior to surgery [31–33]. In our opinion, it makes sense to use anti PD-1 or PD-L1 mAbs prior to surgery as part of the neoadjuvant regimen. In this setting, postsurgical DC vaccinations would boost an already unleashed T-cell response. However, it must be taken into account that metastatic non-MSI colon cancer is mostly refractory to checkpoint inhibitors.

All considered, in spite of the reduced number of cases, results are very encouraging in favor the vaccination arm and indicative of the potential of DC vaccinations with autologous tumor antigens for patients with colon cancer liver metastasis amenable to complete surgical resections.

## Additional files

Additional file 1: (PDF 1991 kb)

Additional file 2: (PDF 1084 kb)
