# Supplementary material for: A randomized phase II clinical trial of dendritic cell vaccination following complete resection of colon cancer liver metastasis
Source: J Immunother Cancer. 2018 Sep 29;6:96. doi: 10.1186/s40425-018-0405-z (PMC6164167; doi:10.1186/s40425-018-0405-z)
Supplement: Supplementary file 1 — (PDF 1991 kb) [file 40425_2018_405_MOESM1_ESM.pdf]

Supplementary File 1

A

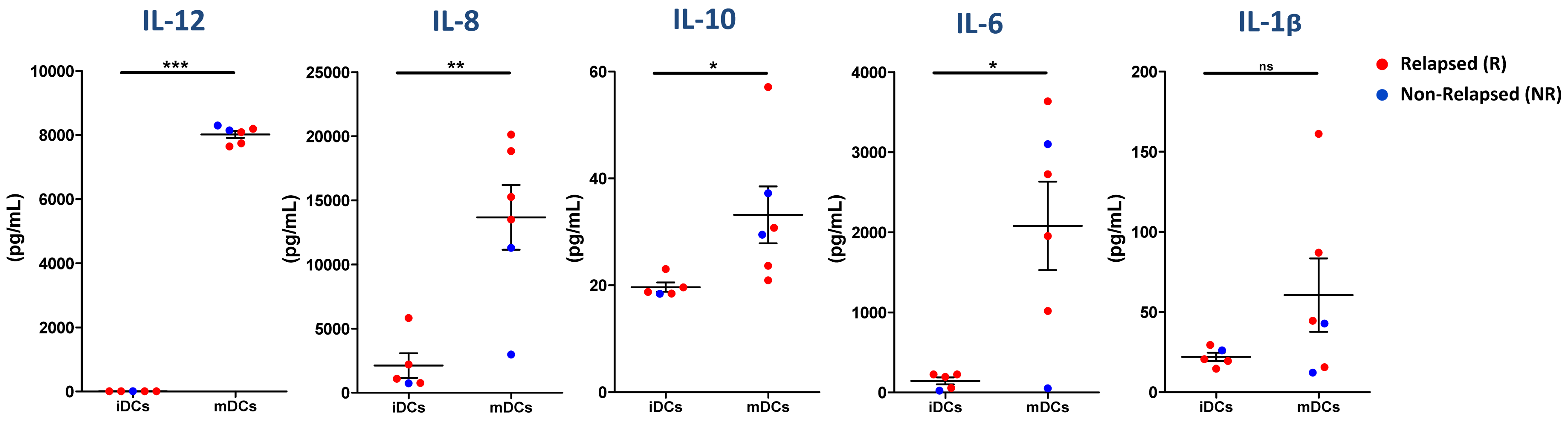

B

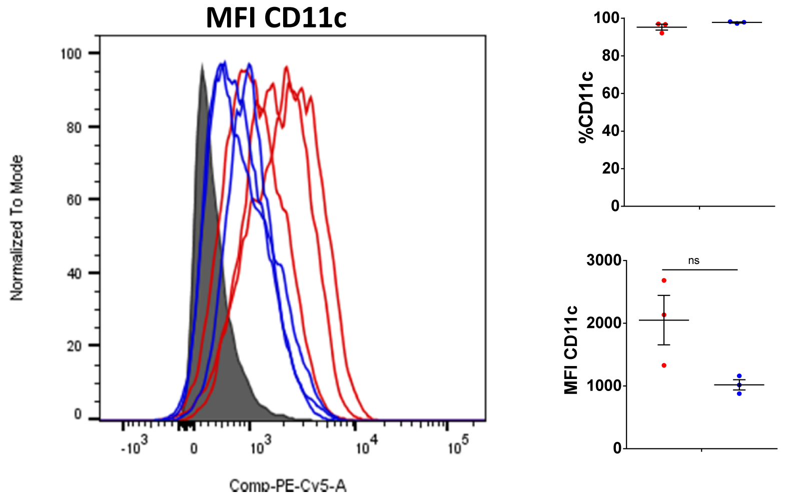

C

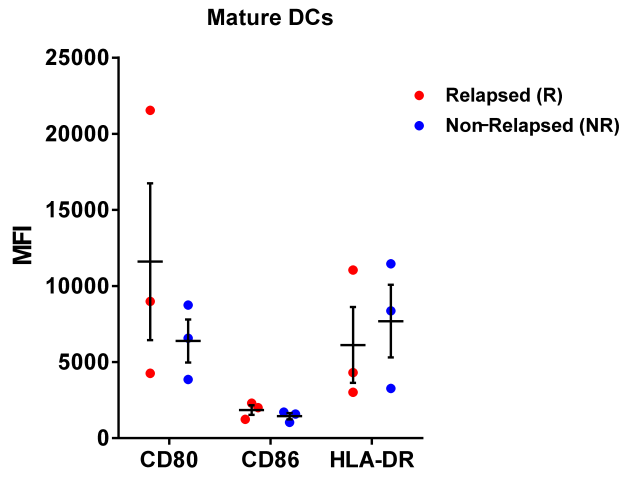

Dendritic cell maturation status of treated patients. Dendritic cell therapy products in six available cryopreserved cases were tested for maturation/activation 48 hours following maturation induction with polyILCL, IFNα and TNFα. (A) Concentration of indicated cytokines in the DC culture supernatants. Dots represent individual patients color-coded for relapsed and non-relapsed cases. (B) Represents FACS staining for CD11c staining % and MFI. (C) Levels of expression of surface maturation markers.
