# Supplementary material for: A randomized phase II clinical trial of dendritic cell vaccination following complete resection of colon cancer liver metastasis
Source: J Immunother Cancer. 2018 Sep 29;6:96. doi: 10.1186/s40425-018-0405-z (PMC6164167; doi:10.1186/s40425-018-0405-z)
Supplement: Supplementary file 2 — (PDF 1084 kb) [file 40425_2018_405_MOESM2_ESM.pdf]

Supplementary File 2

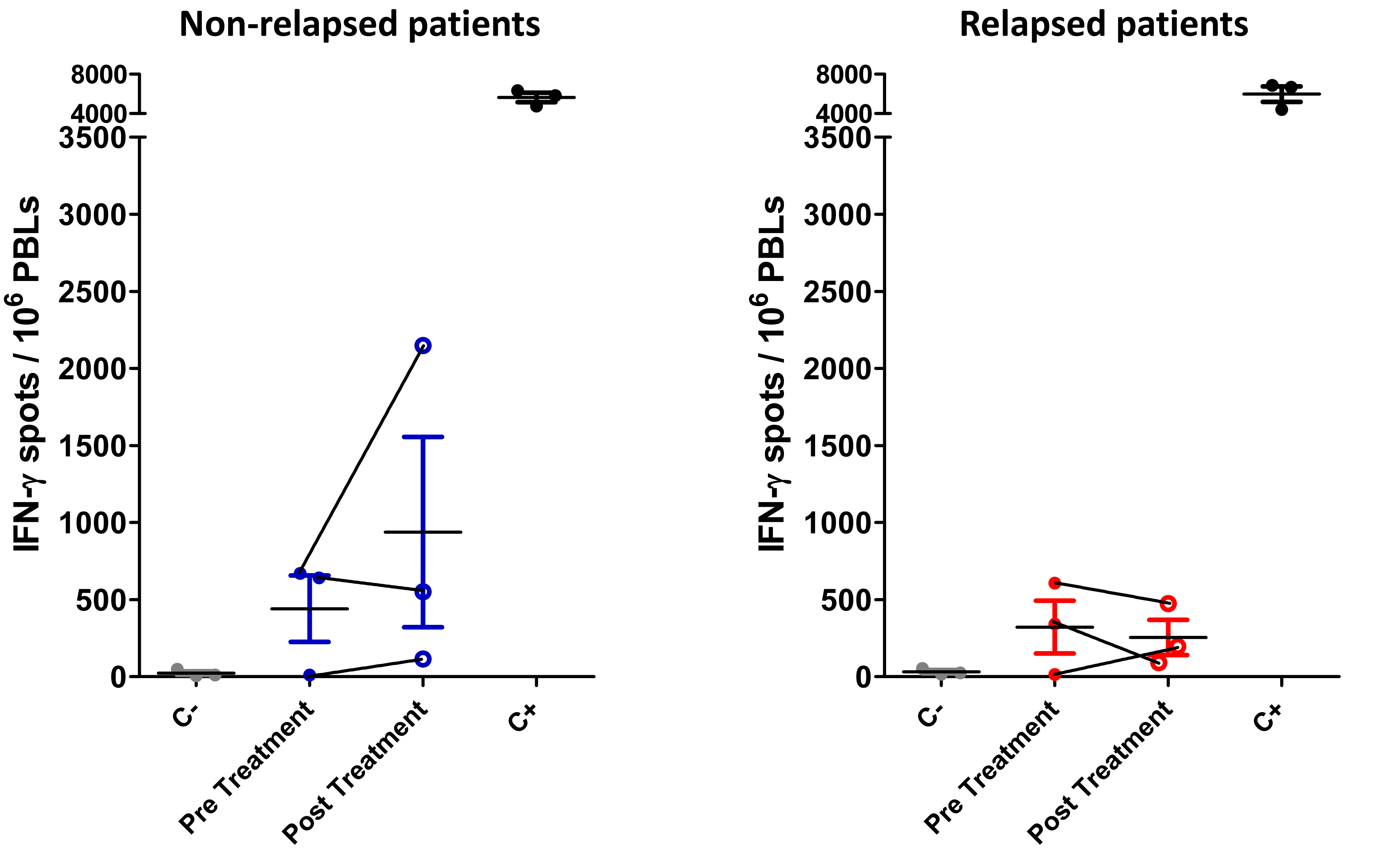

INF-γ ELISPOTS analyses. Frozen DC and sequential PBMC samples were available from six treated cases including three relapsed and three non-relapsed. PBMC obtained pre-treatment (apheresis date) and post-treatment (four weeks after finishing the second treatment cycle) were pulsed with autologous tumor lysate to evaluate IFN-γ-producing spots. Positive control (C+) was SEB and culture medium was used as negative control (C-). Non-relapsed patients are presented on the left graph and relapsed cases on the right graph.
